# Risk Factors in Pediatric Blunt Cervical Vascular Injury and Significance of Seatbelt Sign

**DOI:** 10.5811/westjem.2018.9.39429

**Published:** 2018-10-18

**Authors:** Irma T. Ugalde, Mary K. Claiborne, Marylou Cardenas-Turanzas, Manish N. Shah, James R. Langabeer, Rajan Patel

**Affiliations:** * McGovern Medical School at The University of Texas Health Sciences Center, Department of Emergency Medicine, Houston, Texas; † McGovern Medical School at The University of Texas Health Sciences Center, Department of Diagnostic and Interventional Radiology, Houston, Texas; ‡ McGovern Medical School at The University of Texas Health Sciences Center, The University of Texas Health Sciences Center School of Biomedical Informatics, Houston, Texas; § McGovern Medical School at The University of Texas Health Sciences Center, Department of Pediatric Surgery and Neurosurgery, Houston, Texas; ¶ Phoenix Children’s Hospital, Department of Pediatric Emergency Medicine, Phoenix, Arizona

## Abstract

**Introduction:**

Computed tomography angiography (CTA) is used to screen patients for cerebrovascular injury after blunt trauma, but risk factors are not clearly defined in children. This modality has inherent radiation exposure. We set out to better delineate the risk factors associated with blunt cervical vascular injury (BCVI) in children with attention to the predictive value of seatbelt sign of the neck.

**Methods:**

We collected demographic, clinical and radiographic data from the electronic medical record and a trauma registry for patients less than age 18 years who underwent CTA of the neck in their evaluation at a Level I trauma center from November 2002 to December 2014 (12 years). The primary outcome was BCVI.

**Results:**

We identified 11,446 pediatric blunt trauma patients of whom 375 (2.7%) underwent CTA imaging. Fifty-three patients (0.4%) were diagnosed with cerebrovascular injuries. The average age of patients was 12.6 years and included 66% males. Nearly half of the population was white (52%). Of those patients who received CTA, 53 (14%) were diagnosed with arterial injury of various grades (I–V). We created models to evaluate factors independently associated with BCVI. The independent predictors associated with BCVI were Injury Severity Score >/= 16 (odds ratio [OR] [2.35]; 95% confidence interval [CI] [1.11–4.99%]), infarct on head imaging (OR [3.85]; 95% CI [1.49–9.93%]), hanging mechanism (OR [8.71]; 95% CI [1.52–49.89%]), cervical spine fracture (OR [3.84]; 95% CI [1.94–7.61%]) and basilar skull fracture (OR [2.21]; 95% CI [1.13–4.36%]). The same independent predictors remained associated with BCVI when excluding hanging mechanism from the multivariate regression analysis. Seatbelt sign of the neck was not associated with BCVI (p=0.68).

**Conclusion:**

We have found independent predictors of BCVI in pediatric patients. These may help in identifying children that may benefit from screening with CTA of the neck.

## INTRODUCTION

The incidence of head or neck vascular injury after blunt trauma ranges from 0.03–0.9% of pediatric injured patients.^1^–^4^ Our focus is on blunt cervical vascular injury (BCVI). Without recognition and treatment, BCVI can result in neurologic morbidity or death.^4^–^6^ A challenge in diagnosing BCVI is that symptoms may not initially present with focal, neurologic findings. Studies show there is often a delay in neurologic symptoms up to 10–72 hours after trauma in adults and children alike.^7^,^8^ Screening criteria for adults are well established as described by the Denver and Memphis criteria.^9^ There are no clearly delineated risk factors for pediatric BCVI nor standardized treatments.^10^ The current recommendations of the Eastern Association for the Surgery of Trauma (EAST) recommends that pediatric patients should be screened using the same criteria as those in adult populations.^11^

Due to the often-occult presentation of these injuries and paucity of research until recently, the true incidence, risk factors and treatment regimens in pediatric BCVI are not certain. Yet a more generous screening regimen following the recommendations in adults may be problematic. Children are more susceptible to carcinogenic effects of ionizing radiation and they have a longer life expectancy during which cancer risk accumulates and can manifest.^12^,^13^ The standard radiation dose of a brain and neck computed tomography angiogram (CTA) is about 16.400 millisieverts (mSv) while that of CT of the head is about 2.00 mSv.^14^ A goal in pediatric radiology and trauma is to use dose-reduction strategies to reduce the number of radiation-induced cancers.^15^ To comply with these goals and avoid radiation exposure when not warranted, a good screening algorithm is needed to determine those at high risk of BCVI. There is evidence that decision rules have helped decrease the number of imaging studies in pediatric head trauma.^16^–^18^

The purpose of our study was to determine the incidence of BCVI along with risk factors and treatment regimens observed in the pediatric trauma patients at our Level I trauma center. We also set out to determine the significance of the seatbelt sign in BCVI.

## METHODS

The study was a hospital-based cohort, retrospective review of patients less than 18 years of age in our trauma registry with blunt trauma who had a CTA of the neck performed from November 2002 to December 2014 in our Level I trauma center. We grouped the patients into age groups of less than 15 years and 15 years and older, as well as less than 2 years of age, 2–5 years, 6–14 years, and 15–17 years of age. Among the patients who underwent a CTA of the neck, individual and clinical markers were recorded including age, sex, race, Glasgow Coma Scale Score (GCS), mechanism of injury, Injury Severity Score (ISS), presence of cervical bruit, seatbelt sign of neck, hanging mechanism, focal neurologic exam and presence of laceration. We reviewed adjunct radiographic studies for injuries including cerebral hemorrhage, infarct on head imaging, facial fracture, cervical spine fracture or ligamentous injury, basilar skull fracture, clavicle fracture, thoracic spine fracture, rib fracture, and scapula fracture.

**Population Health Research Capsule**

What do we already know about this issue?

Computed tomography angiography (CTA) can screen patients for blunt cervical vascular injury (BCVI), but pediatric risk factors are not clear. Judicious CTA use is necessary in children.

What was the research question?

What are the risk factors for BCVI in children and is the seatbelt sign a reliable predictor?

What was the major finding of the study?

Some but not all of the risk factors of pediatric BCVI are similar to those in adults. Seatbelt sign is not a predictor.

How does this improve population health?

Findings contribute to evidence to clarify appropriate CTA screening with its inherent radiation exposure, for BCVI in children, limiting the exposure to those at highest risk.

While ISS is not available in the trauma bay during an evaluation we are using it here as a surrogate for degree of severity of trauma, which may be used along with other factors to influence the decision to perform imaging on a patient (See Appendix A for data extraction form). These clinical and radiographic covariates were largely extrapolated from those included in the Memphis, Denver and EAST criteria to assess their significance in children. Data were extracted from the trauma registry and the electronic medical record (EMR) and recorded in a secure database (See Appendix B for trauma registry data abstraction methods). Seatbelt sign represented blunt injury to the neck including deeper abrasions, hematoma, “seatbelt sign” or deep bruising of the neck. We separated mechanisms of injury into three groups: motor vehicle collisions, other motorized vehicles, and other blunt injury. Two physicians each extracted data on all patients independently. Any inconsistencies were reviewed by both and reexamined in the EHR and a conclusion made.

Our outcome of interest was arterial injury of the neck. The images and electronic medical record of the patients found to have BCVI in our cohort were queried for type of vessel damaged and mode of treatment in addition to the parameters mentioned prior. Vascular injuries were characterized as the following: internal carotid artery, common carotid artery, and vertebral artery. Mode of treatment included observation, aspirin, anticoagulation, and surgical intervention, including endovascular stenting and ligation. The observation group included those who had collaterals negating need for intervention, those for whom the positive radiological report was felt to be artifact by the treating physician, or patients for whom observation was the dominant management strategy. This group also included the patients who died within 48 hours of presentation for devastating head injury. Each patient was assigned to the highest grade of injury when more than one lesion was present and to the most aggressive treatment plan received. Observation was the least aggressive treatment plan followed by aspirin, anticoagulation, and finally surgical intervention.

All arterial injuries were graded by a neuroradiologist according to the injury scale proposed by Biffl and colleagues.^19^ In this classification system, grade I injury involved intimal irregularity with < 25% narrowing, grade II injury involved vessel dissection or presence of hematoma with > 25% narrowing, grade III injury indicated pseudoaneurysm, grade IV represented vessel occlusion, and grade V represented transection of the vessel with extravasation.^19^

The pediatric trauma CTA scanner used during the study period was a Siemens Sensation 40 Helical CT (40 slice) and a Siemen’s Definition AS+ Helical CT (128 slice). There is no difference in diagnostic utility of cervical vascular injury above the 16-slice scanners; thus, the ones used in our study were equivalent.

### Statistical Analysis

We report on frequencies, proportions, and measures of central tendency. Factors associated with BCVI were analyzed using univariate analyses. Ordinal and nominal variables are reported using the chi square test or Fisher’s exact test if the count in the contingency table was ≤6. Continuous variables were analyzed using Student’s t-test. The logistic regression model includes all clinical variables and age. The covariates initially included the following: age, GCS, ISS, cerebral hemorrhage, seatbelt sign, infarct on head imaging, hanging mechanism, mechanism of injury, presence of facial fractures, cervical spinal fractures, basilar skull fractures, clavicle fractures, thoracic fractures, rib fractures, and scapula fractures. We used the backward stepwise elimination (Wald) approach to determine the covariates included in the final model. The functionality of the adjusted final model is verified, and the Hosmer and Lemeshow goodness-of-fit test is reported. Due to the increased standard error and the wide confidence interval (CI) of the covariate hanging mechanism, we created a second model excluding this particular covariate. A *p*-value <.05 (two-tailed) was considered statistically significant in all tests. We performed all analyses with IBM SPSS software (version 23).

## RESULTS

During the study period, 13,735 pediatric trauma patients less than 18 years of age were evaluated in the emergency department and entered into the trauma registry. Of these, 11,446 suffered blunt injuries (83.3%). The 375 (3.3%) children who experienced blunt trauma and underwent screening with neck CTA were included in this study. Fifty-three patients were diagnosed with cervical vascular injuries (0.5% of all pediatric blunt trauma patients evaluated in the study period; 14% of all blunt trauma patients screened with CTA). Some of these patients had more than one vascular lesion. The mean age of patients was 12.6 years. Non-Whites (48%) and Whites (52%) were nearly equally distributed.

Univariable factors associated with cervical vascular injury (p< 0.05) were GCS </= 8, ISS >/= 16, presence of cerebral hemorrhage, infarct on head imaging, cervical spine fracture and basilar skull fracture. Seatbelt sign was not associated with cervical vascular injury (p=0.68) (Table 1). We created two models of multivariate logistic regression. Model 1, including the covariate hanging mechanism, showed the factors independently associated with cerebral vascular injury were ISS >/= 16 (odds ratio [OR] [2.35]; 95% CI [1.11–4.99%]), infarct on head imaging (OR [3.859]; 95% CI [1.49–9.93%]), hanging mechanism (OR [8.71]; 95% CI [1.52–49.89%]), cervical spine fracture (OR [3.84]; 95% CI [1.94–7.61%]) and basilar skull fracture (OR [2.21]; 95% CI [1.13–4.36%]) (Table 2). The goodness of fit test for this first model had a chi square= 8.37, degrees of freedom = 5, and p-value= 0.14. When we excluded hanging mechanism from the analysis, our model 2 had a better goodness-of-fit test (chi square = 5.57, degrees of freedom = 5, and p-value= 0.32) (Table 3). Importantly, the independent factors associated with BCVI in the first model remained the same.

**Figure 1ab:**
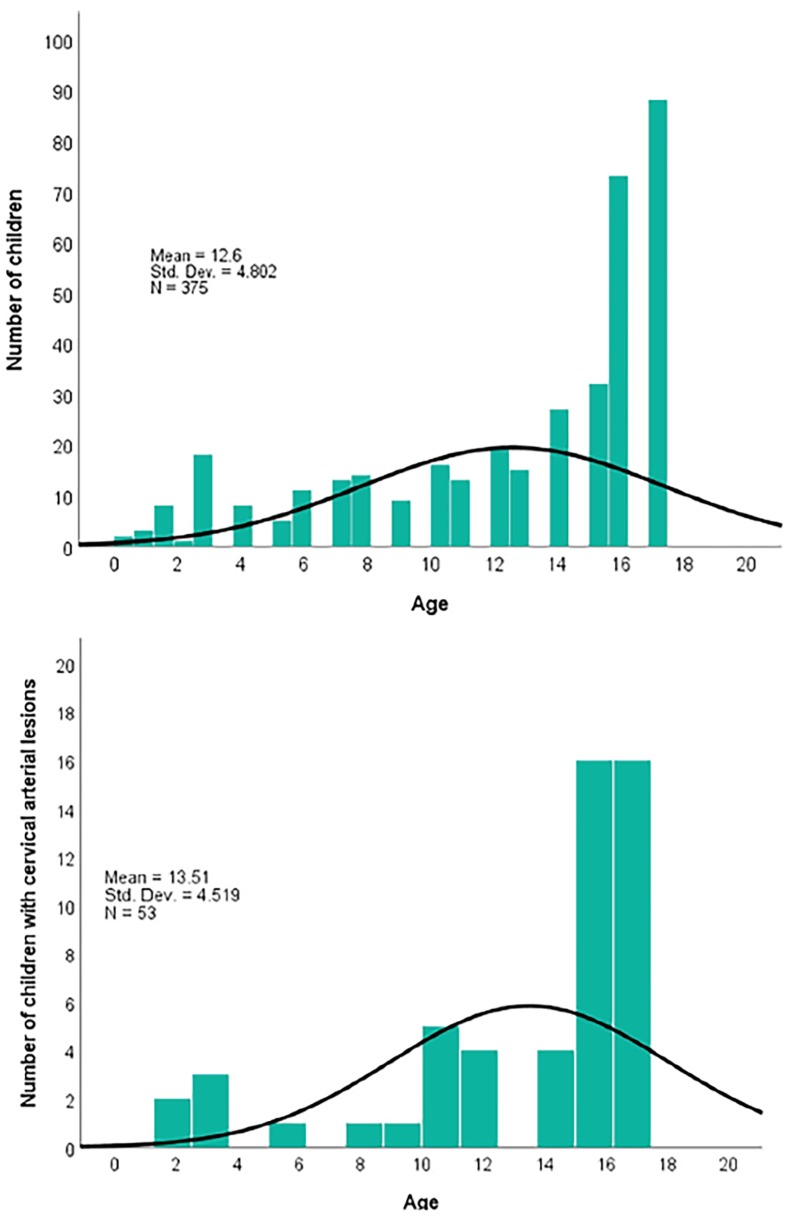
a) Histogram of ages of the total sample of children included in the study (top); b) Histogram of ages of children with cervical arterial lesions (bottom).

There were similar proportions of patients in the less-than-15 years of age (182, 45.8%) group as compared to the 15 and older group (193, 51.5%). When we further separated the groups, there were 44/375 (12%) patients in the five years old and under group. See Figure 1A and 1B for a histogram with distribution of all ages in this cohort and a distribution of ages of children with vascular injuries, respectively. Ninety one percent of the vascular injuries were found in the six years and older group while only nine percent of vascular injuries were found in the preschool age group (five years and under). More than half of all lesions (32) were found in the 15 years and older group; 17% of this group were found to have BCVI.

There was a total of 63 cervical vascular lesions identified within the 53 patients, since some patients had more than one vessel injured (i.e., eight patients had two vessels involved, and one had three vessels injured). In our sample, the majority of the lesions were grade I (34%) followed by grade II and IV lesions (23% each). Grade III injuries composed 21% of the group. There were no grade V lesions in our sample.

**Figure 2:**
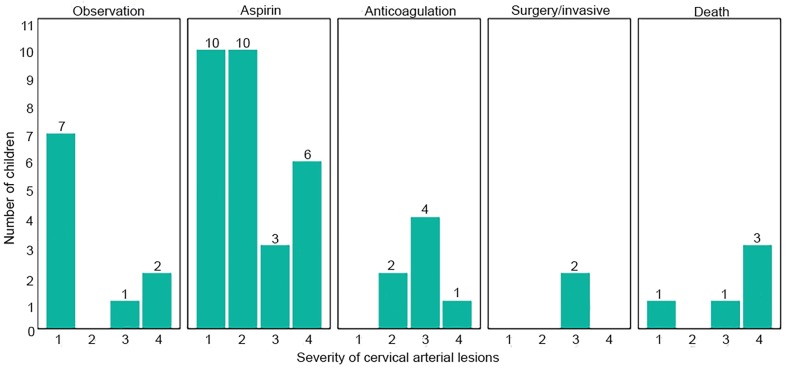
Types of treatments and mortality associated with graded severity of vascular lesions for study cohort, Grade I–IV. No Grade V was observed in this study.

Medication management was the most common treatment plan. Aspirin or anticoagulation was used for 66% of all patients and varied by the lesion grade. Grade I lesions tended to receive aspirin only, while grades II and higher included the use of anticoagulants. An interventional approach was used for only 1.8% of patients. In-hospital mortality occurred in 11% of the cases. Six patients expired within 48 hours of presentation to the ED with five of these dying within 24 hours. All of the deaths were attributed to brain injuries. Of these, one patient had cerebral edema and two others had ischemic changes on CT brain images. Figure 2 shows the number of children per graded lesion and the type of management received in each category.

## DISCUSSION

Our large retrospective review identified similarities and differences in risk factors for BCVI in children when considering the risk factors identified in adults. Over the last decade there have been increased efforts to discover clinical risk factors for BCVI, specifically in children, but with little consensus. In 1999 Lew et al. reviewed the National Pediatric Trauma Registry with 57,659 pediatric blunt trauma patients and found that clavicular fracture demonstrated the strongest association with blunt carotid artery injury.^20^ While we included clavicular fracture in our series as a potential risk factor, it did not bear an association with BCVI, with only four clavicular fractures found in the BCVI group and 34 in those without BCVI.

In another series, Jones et al. found that more than two thirds of pediatric patients presenting with stroke did not have screening indications similar to those observed in the adult protocols.^2^ These researchers found a high percentage of blunt cerebrovascular injuries with cervical spine injuries in 19/45 (40%) of patients.^2^ We also found cervical spine injuries to be significantly associated with BCVI in our cohort. This association makes sense from a mechanical and anatomical view as both cervical spine injuries and BCVI result from similar etiologies such as cervical hyperextension and rotations, hyperflexion, or a direct blow. Intimal disruption from the trauma causes emboli or occlusion of the vessel. As the cervical vasculature is adjacent to the cervical spine, if the latter is injured, the former is at risk for injury as well. In contrast, Kopelman and his group identified only 11 pediatric patients with documented BCVI, with 91% of these patients having a risk factor that had been associated with BCVI in adult populations. They concluded that the risk factors for pediatric BCVI mimic those of the adult population.^3^ While some adult risk factors are confirmed in our series, others were not associated with BCVI in children such as facial fractures, Le Fort fractures, and chest injury such as rib, scapula, thoracic and clavicle fractures.

Ravindra and colleagues identified 234 patients who had screening for blunt cerebrovascular injuries with 37 injuries observed. They determined that fracture through the carotid canal, petrous temporal bone fracture, GCS < 8, focal neurological deficit, and stroke on initial CT were independent factors predicting vascular injuries, which do parallel some of our findings.^10^ When validated in a multicenter trial, the sensitivity of the Utah score remained low at 59%, rendering a questionable utility of the score as an initial screening tool.^10^ With the addition of mechanism of injury to this score, the McGovern-Utah score brings the sensitivity up to 81%.^21^ While promising, this score still needs validation with other populations. Furthermore, cerebrovascular injuries of both head and neck were included in these cohorts.

We focused our study on CTAs of the neck and specifically analyzed the impact of seatbelt sign as a marker for vascular injury. Our study did not find seatbelt sign to be an independent factor associated with BCVI, which supported previous studies’ findings, albeit other reports included smaller sample sizes than our study. In 2014, Desai found that the cervical seatbelt sign was not associated with BCVI in children.^5^ Dhillon and colleagues found a weak correlation between the cervical seatbelt sign and vascular injury in a mainly adult population.^22^ These authors concluded that a protocol for CTA of the neck for patients with a cervical seatbelt sign can be reserved for those with associated injuries on physical exam and/or findings on standard trauma imaging.^22^

The EAST also recommends against the use of seatbelt sign to independently select patients for screening, although in practice it is sometimes still used.^11^ A possible explanation for the lack of significance of the seatbelt sign in our current study is that we included records for only those patients who had a CTA neck performed, and it is possible that a child with seatbelt sign of the neck did not receive imaging. If imaging was not performed and the same patient followed up at a different institution with a vascular injury, a patient would have been missed in our cohort. Furthermore, due to its retrospective design, physical exam findings may not be documented accurately or simply not included. In addition, due to our interest in the exam findings of the neck, we focused only on CTA neck in this study. In a different study at our institution we included both CTA neck and brain to develop the McGovern score.^21^

## LIMITATIONS

The retrospective design with inherent recall bias and the inclusion of a single institution are limitations. Our Level I trauma center is located in the inner city and our patient body is composed of a large percentage of uninsured and minority groups and may not be generalizable to all populations. Furthermore, with the collection of data covering a 12-year span, there may have been differences in practice patterns guiding the screening of BCVI.

It is also reasonable to hypothesize that our incidence of blunt CVI may have been higher if all blunt trauma patients (11,446) had been screened with a CTA of the neck as a large number were asymptomatic. It is quite possible that asymptomatic Grade I or II lesions did not go on to develop symptoms and were unreported without imaging. Yet it is not feasible to screen all trauma patients due to monetary costs and radiation risk to our pediatric patients. Moreover, we chose CTA as our screening vs. other modalities such as magnetic resonance angiogram (MRA) and angiogram because in the ED setting, CTA is the most logical choice for quick diagnoses. Research regarding the difference in diagnostic accuracy of CTA and MRA in blunt cerebrovascular injury has been mixed, but in recent years CTA has emerged as the study of choice, replacing the four-vessel digital subtraction angiography.^23^–^25^ It is possible our facility may have had patients transferred from outside institutions or worked up during their hospital stay with other imaging modalities not included in our analysis. We focused on patients with acute trauma presenting in the ED; thus, this constricted sample of only patients imaged for inclusion in the study is a limitation that may be accounted for in a prospective trial.

While most of our predictors of injury had narrow CIs, two in particular had wider ones. We postulate that the two factors with the widest CIs – infarct on head imaging and hanging mechanism – were affected by the small sample size of patients with these findings. There were 25 (6.7%) patients with infarct on head imaging and eight (2.1%) patients with hanging mechanism in the cohort of 375 patients. An even larger, prospective multicenter trial might validate our findings. Both these are risk factors in adult BCVI and our cohort.

There is no standard of care on selecting the optimal treatment when BCVI is discovered and no set pathway to mitigate risk of cerebrovascular accident. The majority of our cohort was treated conservatively or with aspirin alone, and only one out of 53 patients received surgical intervention. Our study did not have enough power to compare the effectiveness of different treatment plans, but we did not find adverse events related to either method in our series. The prevalence as well as the short- or long-term effects of adverse events is a topic that would benefit from research focused on the risks and benefits of treatments.

Further work should also delve into the true risk of stroke in the pediatric population. While we did not look at this specifically, it is information that deserves attention in future prospective studies. The pathophysiology of stroke may be different in children and adults. Children have greater elastic resilience of their vessels than adults and have more elastic bone and soft tissues around the vessels that can potentially absorb the kinetic energy of high-impact blunt trauma better than adults. Less diseased vessels in children may also allow for quicker recovery time in the setting of injury. There is evidence to suggest that BCVI in adults is more severe than in children.^26^ It is true that the treatment of graded lesions vary by institution; thus, is not standardized. We believe a more standardized approach in diagnosing cervical vascular lesions may pave the way for more research into treatment and outcomes.

The need and interest to develop pediatric guidelines for CTA screening is demonstrated by the recent flurry in publications on this topic. Our study specifically extrapolated risk factors for BCVI in a pediatric population in one of the largest cohorts to date. It should be noted that clinical judgment may trump clinical guidelines, but we are in need of developing a robust rule. A prospective, multi-site, observational study is needed to devise a screening tool that is accurate enough to capture patients at risk for BCVI.

## CONCLUSION

We identified independent predictors of cervical vascular injury in children in one of the largest samples to date – namely ISS >/= 16, presence of cerebral hemorrhage, infarct on head imaging, cervical spine fracture, and basilar skull fracture. These factors may help raise awareness and improve the quality of care of children undergoing a trauma evaluation for possible screening with CTA.

## Supplementary Information





## Figures and Tables

**Table 1:** Univariate analyses of demographic and clinical factors associated with blunt cervical vascular lesions in children.

Characteristic	All N=375 (100%)	Cervical vascular lesion N=53 (14.1%)	No cervical vascular lesion N=322 (85.9%)	p-value
Demographic factors				
Age				
<15 years old	182 (45.8)	21 (39.6)	161 (50)	0.16
≥15 years old	193 (51.5)	32 (60.4)	161 (50)	0.16
Age				
<2 years old	7 (1.9)	1 (1.9)	6 (1.9)	0.57
2–5	37 (9.9)	4 (7.5)	33 (10.2)	0.57
6–14	138 (36.8)	16 (30.2)	122 (37.9)	0.57
15–17	193 (51.5)	32 (60.4)	161 (50)	0.57
Sex				
Male	246 (65.6)	29 (54.7)	217 (67.4)	0.07
Female	129 (34.4)	24 (45.3)	105 (32.6)	0.07
Race				
White	193 (51.6)	34 (64.2)	159 (49.5)	0.05
Non-White	181 (48.4)	19 (35.8)	162 (50.5)	0.05
Clinical factors				
GCS				
≤8	126 (33.6)	27 (50.9)	99 (30.7)	0.004
>8	249 (66.4)	26 (49.1)	223 (69.3)	0.004
ISS				
<16	173 (46.1)	13 (24.5)	160 (49.7)	0.001
≥16	202 (53.9)	40 (75.5)	162 (50.3)	0.001
Cerebral hemorrhage				
Yes	178 (47.5)	33 (62.3)	145 (45)	0.02
No	197 (52.5)	20 (37.7)	177 (55)	0.02
Seatbelt sign of neck				
Yes	86 (22.9)	11 (20.8)	75 (23.3)	0.68
No	289 (77.1)	42 (79.2)	247 (76.7)	0.68
Infarct on head CT				
Yes	25 (6.7)	10 (18.9)	15 (4.7)	≤0.001
No	350 (93.3)	43 (81.1)	307 (95.3)	≤0.001
Hanging mechanism				
Yes	8 (2.1)	3 (5.7)	5 (1.6)	0.09*
No	367 (97.9)	50 (94.3)	317 (98.4)	0.09*
Mechanism				
MVC	212 (56.5)	31 (58.5)	181 (56.2)	0.58
Other motorized	80 (21.3)	13 (24.5)	67 (20.8)	0.58
Other blunt	83 (22.1)	9 (17)	74 (23)	0.58
Facial fractures				
Yes	103 (27.5)	17 (32.1)	86 (26.7)	0.42
No	272 (72.5)	36 (67.9)	236 (73.3)	0.42
Cervical spinal fracture				
Yes	86 (22.9)	23 (43.4)	63 (19.6)	≤0.001
No	289 (77.1)	30 (56.6)	259 (80.4)	≤0.001
Basilar skull fracture				
Yes	127 (33.9)	26 (49.1)	101 (31.4)	0.01
No	248 (66.1)	27 (50.9)	221 (68.6)	0.01
Clavicle fracture				
Yes	35 (9.3)	4 (7.5)	31 (9.6)	0.80*
No	340 (90.7)	49 (92.5)	284 (90.4)	0.80*
Thoracic fracture				
Yes	50 (13.3)	10 (18.9)	40 (12.4)	0.20
No	325 (86.7)	43 (81.1)	282 (87.6)	0.20
Rib fracture				
Yes	65 (17.3)	8 (15.1)	57 (17.7)	0.64
No	310 (82.7)	45 (84.9)	258 (82.3)	0.64
Scapula fracture				
Yes	16 (4.3)	2 (3.8)	14 (4.3)	0.99*
No	359 (95.7)	51 (96.2)	308 (95.7)	0.99*

*IQR,* interquartile range; *GCS*, Glasgow Coma Scale Score; *ISS*, injury Severity Score; *CT*, computed tomography; MVC, motor vehicle collision.

* Fisher’s exact test.

**Table 2:** Model 1: Multivariate logistic regression analysis of clinical factors associated with cervical vascular lesions, including the covariate hanging mechanism.

	Variables in the equation							

	B	SE	Wald	df	p-value	OR	95% CI for OR	

							Lower	Upper
ISS ≥16	0.857	0.383	5.007	1	0.02	2.35	1.11	4.99
Infarct on head CT	1.348	0.484	7.761	1	0.005	3.85	1.49	9.93
Hanging mechanism	2.165	0.890	5.911	1	0.015	8.71	1.52	49.89
Cervical spinal fracture	1.346	0.349	14.907	1	0.000	3.84	1.94	7.61
Basilar skull fracture	0.796	0.345	5.318	1	0.02	2.21	1.13	4.36
Constant	−3.303	0.370	79.691	1	0.000	0.04		

*ISS,* injury severity score*; CI,* confidence interval; *OR*, odds ratio; *CT*, computed tomography; *B*, beta; *SE*, standard error; *df*, degrees of freedom.

**Table 3:** Model 2: Multivariate logistic regression analysis of clinical factors associated with cervical vascular lesions, excluding the covariate hanging mechanism.

	Variables in the equation							

	B	SE	Wald	df	p-value	OR	95% CI for OR	

							Lower	Upper
ISS ≥16	.775	.371	4.371	1	.04	2.17	1.05	4.49
Infarct on head CT	1.375	.471	8.531	1	.003	3.95	1.57	9.95
Cervical spinal fracture	1.289	.341	14.289	1	.000	3.63	1.86	7.08
Basilar skull fracture	.746	.339	4.841	1	.03	2.1	1.08	4.1
Constant	−3.148	.352	79.855	1	.000	.04		

*ISS,* injury severity score*; CI,* confidence interval; *OR*, odds ratio; *CT*, computed tomography; *B*, beta; *SE*, standard error; *df*, degrees of freedom.
