# Supplementary material for: Risk Factors in Pediatric Blunt Cervical Vascular Injury and Significance of Seatbelt Sign
Source: West J Emerg Med. 2018 Oct 18;19(6):961–9. doi: 10.5811/westjem.2018.9.39429 (PMC6225950; doi:10.5811/westjem.2018.9.39429)
Supplement: Supplementary file 1 [file wjem-19-961-s001.docx]

Appendix A.

| MRN |
| --- |
| Age |
| Sex |
| race |
| race name |
| **vascular Injury y/n** |
| GCS |
| ISS |
| Focal neurologic deficit Y/N |
| Cerebral hemorrhage Y/N |
| Cervical bruit Y/N |
| Seat belt sign/hematoma/abrasion/soft tissue injury Y/N |
| infacrt on head image Y/N |
| Puncture wound, laceration, penetrating Y/N. |
| Facial fractures Y/N |
| hanging mechanism Y/N |
| cervical spinal fracture Y/N |
| Basilar skull fracture Y/N |
| Mechanism |
| Clavicle Y/N |
| Thoracic Y/N |
| Rib Y/N |
| Scapula Y/N |
