# Supplementary material for: Risk Factors in Pediatric Blunt Cervical Vascular Injury and Significance of Seatbelt Sign
Source: West J Emerg Med. 2018 Oct 18;19(6):961–9. doi: 10.5811/westjem.2018.9.39429 (PMC6225950; doi:10.5811/westjem.2018.9.39429)
Supplement: Supplementary file 2 [file wjem-19-961-s002.docx]

Appendix B

**MEMORIAL HERMANN HOSPITAL**

**TRAUMA SERVICES**

1. **Trauma Registry Process**
2. Daily ER Trauma Log and Transfer Log are printed daily.

This enables us to capture cases that came thru the emergency room and transfers that are direct admit.

1. Trauma Registrar assigned for that day, identify cases that meet the inclusion criteria.
2. Cases are assigned trauma registry number and entered in the trauma registry database with 65 initial data fields such as demographics, times/dates and vitals
3. The charts are then abstracted for prehospital data, diagnoses, procedures, coding, comorbidities complication, outcomes and financial data. The chart is considered complete/closed at after all the above data are abstracted.

Source: EMS Run sheets, Electronic Medical Records, Scanned documents.

1. Deaths are not closed until the autopsy reports are available. We are able to obtain 100% of our autopsy report request from the Medical Examiners’ Office.

1. **Data Validation**
2. 5-10% of the records abstracted by each Trauma Registrar (7 cases per registrar/month) are being audited by the Trauma Registry Manager, PI Nurses and Trauma Program Managers. Any data quality issues are being discussed with the registrar that initially abstracted/closed the record.

This process allows us to identify areas that need to be addressed thru education of the team.

1. Inter/intra rater reliability.

We started this process early 2008, wherein the Trauma Registry Manager pick a cases that each Trauma Registrar will abstract and code individually using a standard abstraction form with specific data points on it. The case is then discussed during monthly educational meeting.

If there are diagnoses coding (AIS, ISS) issues that we ourselves could not agree/resolve using the coding manual, we then send the issues to AAAM (American Association of Automotive Medicine), the owner/authorized agency for AIS (Abbreviated Injury Scale) scoring.

1. The Trauma Registry Manager does a spot check when running reports and discusses issues with each registrar as needed.
2. Software related validation

The trauma registry database (Traumabase- Clinical Data Management) has a built in validation and data cleaning process for both the State and NTDB/TQIP that checks on every field in the database except for some text fields which are very few. We receive regular updates from our software vendor.

Note: We are required to submit data to the State of Texas Trauma Registry, NTDB/TQIP and Regional Registry quarterly. These entities strictly require data to pass their validation process.

Any validation issues identified are being discussed with the registrars.

1. **Education/Certification**
2. We send our registrars to classes (such as, AIS 2005 version), a two day workshop on AIS-ISS coding and scoring, where they test their injury abstraction/coding skills.  Our registrars, once they meet the requirements, are encouraged to take the following certification exams: ATSRCS- CSTR, AAAM-CAISS.

CSTR: Certified Specialist in Trauma Registry (American Trauma Society)

CAISS: Certified Abbreviated Injury Scale Specialist (AIS Certification Board)

As of this time five (5) of nine (9) registrars are CSTR, seven (7) of nine (9) are CAISS.

1. Trauma Registrars are also sent to ICD9/ICD10 Coding classes, ATS Trauma Registry Class or State sponsored Trauma Registry Classes.
2. Regular attendance to the Annual TQIP Conference.
3. Trauma Registrars are required to participate in the following educational offerings: Annual TQIP online Course, Monthly TQIP Quizzes and Webinars.
4. We dedicate one day a month for trauma registrars education which includes topics discussed in the TQIP monthly webinar and TQIP Quiz to basically have a better and consistent understanding of the data dictionary.
5. During our monthly education, we do invite faculties to talk about topics and during these interactions we are able to communicate the importance of documentation in relation to consistent and more specific coding.
6. We maintain a change log folder so we can keep tract on the changes, updates made in the trauma registry database.
7. **Projects**

Our team in partnership with our clinical software analysts has been working on import of data from the electronic medical record. In fact we have completed phase 1 where we are able to pull the data points for initial log entry.

We are now working on phase 2 and looking at more data pull. This process allows consistency and minimizes typographic/human error on some data that are readily available from the emergency medical record.
